# Diagnostic challenges in synchronous ovarian metastasis from rectal cancer: A case report

**DOI:** 10.1097/MD.0000000000017782

**Published:** 2019-11-01

**Authors:** Federico Biolchini, Carolina Castro Ruiz, Erica Pavesi, Giovanni Musci, Maurizio Zizzo, Lara Ugoletti, Valerio Annessi

**Affiliations:** a Department of General Surgery, Chirurgia Area Nord; b Pathology Unit; c Surgical Oncology Unit, Azienda Unità Sanitaria Locale-IRCCS di Reggio Emilia, Reggio Emilia, Italy.

**Keywords:** diagnosis, immunohistochemistry, ovarian metastasis, rectal cancer, treatment

## Abstract

**Introduction:**

Ovarian metastases from rectal cancer are infrequent; thus it might be hard to diagnose and treat them. Our study introduces a challenging case which highlights our method in addressing such an issue.

**Patients concerns:**

A 74-year-old woman was admitted to our Unit showing abdominal pain, vomit, and a gross abdominal mass located in the right iliac fossa and mesogastrium. Oncological markers recorded following abnormalities: carbohydrate antigen 19.9 (Ca19.9) = 453.40 U/mL, carbohydrate antigen 125 (Ca125) = 88.3 U/mL.

**Diagnosis:**

Such a metastatic tumor being difficult to diagnose, we could not achieve a precise preoperative diagnosis. We entered the operating room with a histologic diagnosis that was highly suspicious of colon adenocarcinoma. During surgery, frozen section analysis was positive for primary ovarian cancer. Thanks to the immunohistochemistry test on the histologic specimen, which might be very helpful in diagnosing such metastatic tumor, final pathology report documented ovarian metastasis from rectal cancer.

**Interventions:**

We performed total hysterectomy with bilateral salpingo-oophorectomy and low anterior resection of the rectum with a terminal colostomy. Adjuvant chemotherapy was administered for 6 months using FOLFOX plus panitumumab in first-line therapy.

**Outcome:**

At 8 months from surgery, during follow-up, a local pelvic progression of disease was detected, leading to second-line chemotherapy treatment.

**Conclusion:**

Correct differential diagnosis between primary and metastatic ovarian tumors is paramount in choosing the best treatment which leads to the best possible outcome. In ovarian metastatic tumors, immunohistochemistry could represent an optimal diagnostic tool.

## 1 Introduction

Rectal cancer has a tendency to metastasize into thoracic organs and nervous system, whereas metastases in peritoneum (ovaries included) are less frequent. Nonetheless, when compared with generic adenocarcinomas, mucinous and signet ring adenocarcinomas show a more frequent trend to metastasize within the peritoneum,^[1]^ with a 3% to 14% estimated incidence of ovarian metastasis from colorectal cancer.^[2,3]^

Secondary neoplasms to the ovary, in particular, metastatic colorectal carcinomas of mucinous origin, can represent a diagnostic challenge, as they can mimic primary mucinous carcinoma of the ovaries.^[4,5]^ Correct diagnosis before surgery is crucial to the treatment approach. At present, limited data help define an appropriate treatment of ovarian metastatic disease from colon cancer. Moreover, the consequences on the prognostic impact of metastasectomy are still unknown.^[6]^ Our study introduces a challenging diagnostic case of rectal cancer with metastasis to the ovary.

Our case report was written according to CARE guidelines.^[7]^ Patient informed consent was obtained for the publication of the study.

## 2 Case report

A 74-year-old woman with no comorbidities and without any prior surgery came to our Unit with abdominal pain, vomit, and without any fever. Physical examination revealed a gross abdominal mass in the right iliac fossa and mesogastrium. Blood tests recorded 13.5 nL white blood cell count with a neutrophilic prevalence (453.40 U/mL high carbohydrate antigen 19.9 [Ca19.9] levels, and 88.3 U/mL carbohydrate antigen 125 [Ca125]), whereas AFP and carcinoembryonic antigen (CEA) were negative. Inflammatory markers, such as C-reactive protein and erythrocyte sedimentation rate, were altered.

**Figure 1:**
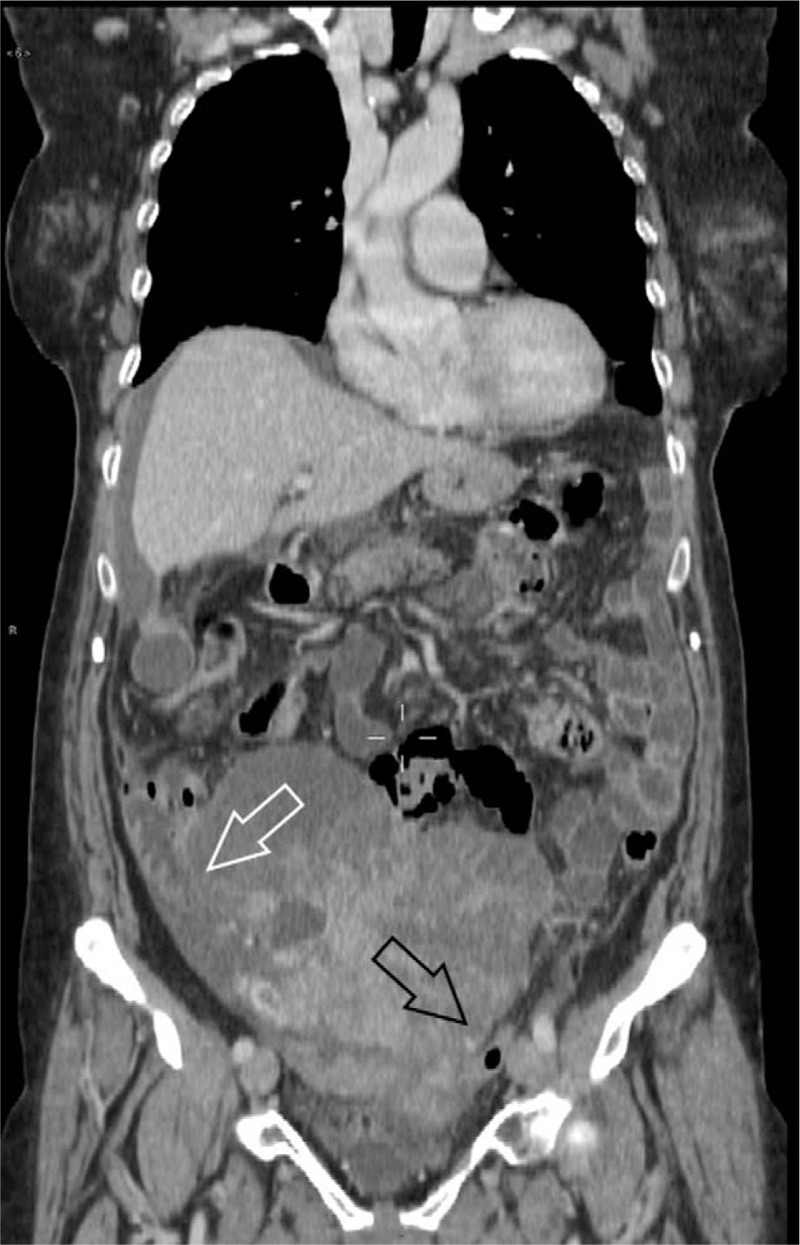
Computed tomography (CT) scan. Inhomogeneous mass tightly attached to the right (white arrow) and sigmoid colon (black arrow).

**Figure 2:**
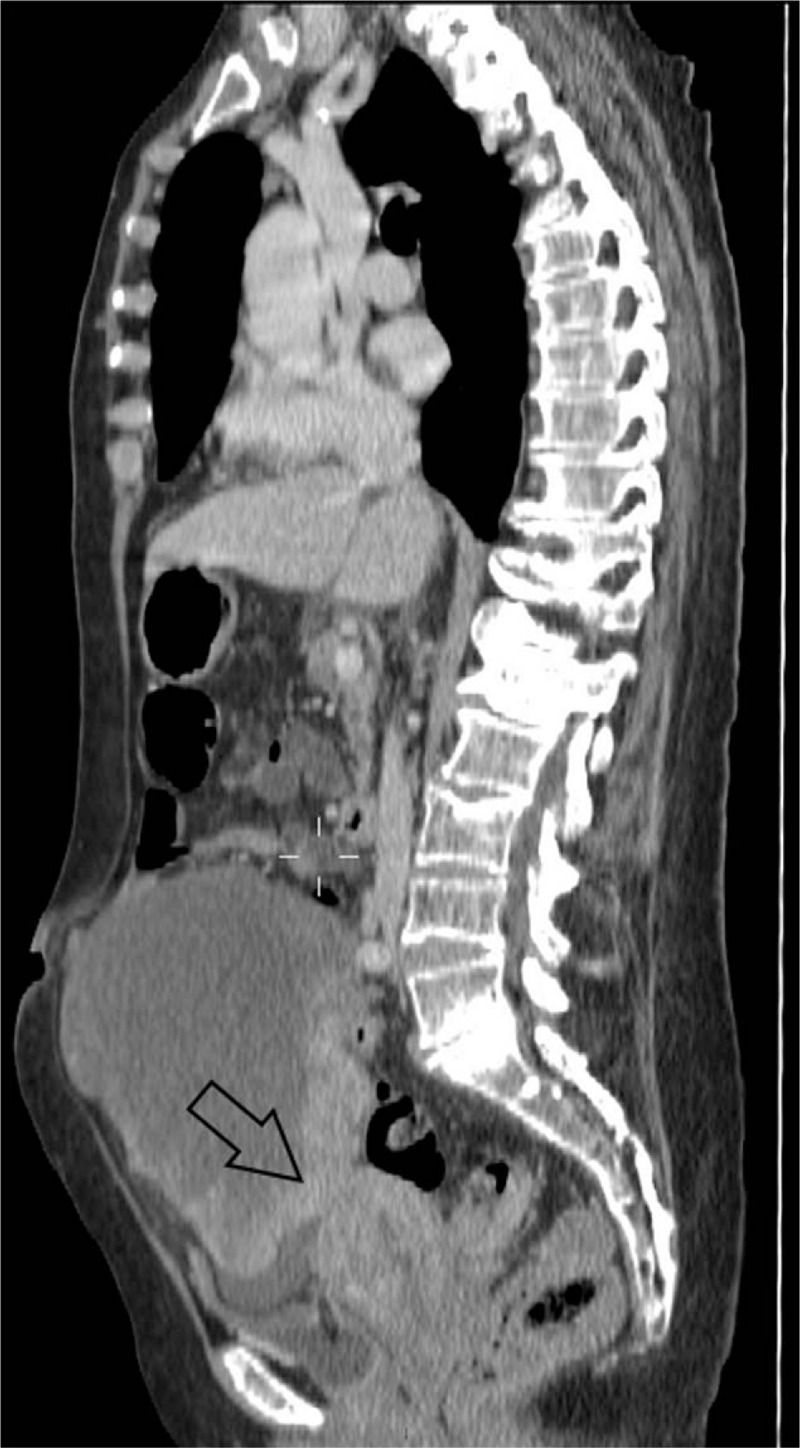
Computed tomography (CT) scan. Loss of the fatty plane between the mass and the uterus (black arrow).

**Figure 3:**
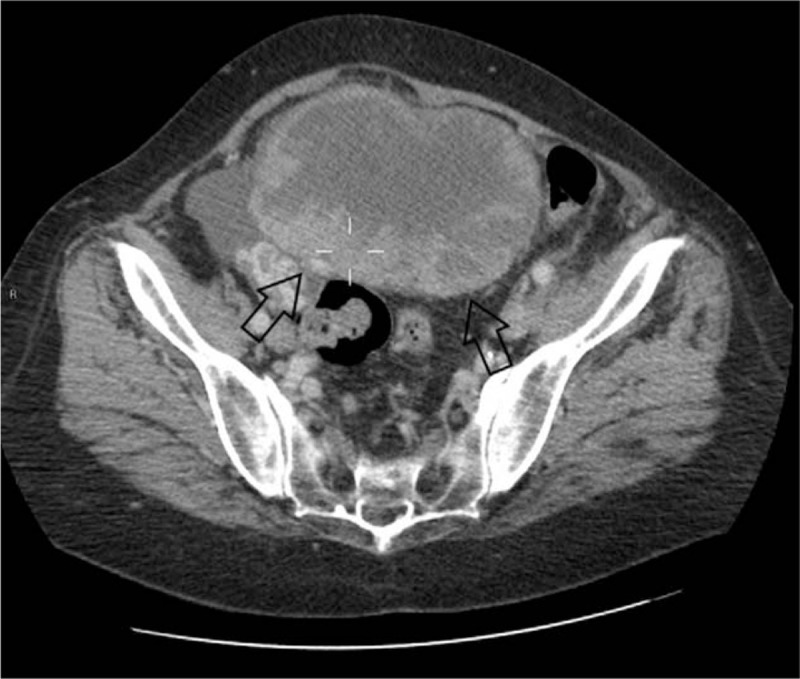
Computed tomography (CT) scan. Presence of peripheral enhancement (black arrows).

Abdomen and pelvis CT detected an inhomogeneous 18 cm × 12.4 cm solid mass with peripheral enhancement, located in the right iliac fossa. After contrast washout, the mass showed the same uterus density. The mass was tightly attached to the right and sigmoid colon with an apparent loss of the fat planes between these organs. We could not rule out an ovarian origin (Figs. 1–3).

**Figure 4:**
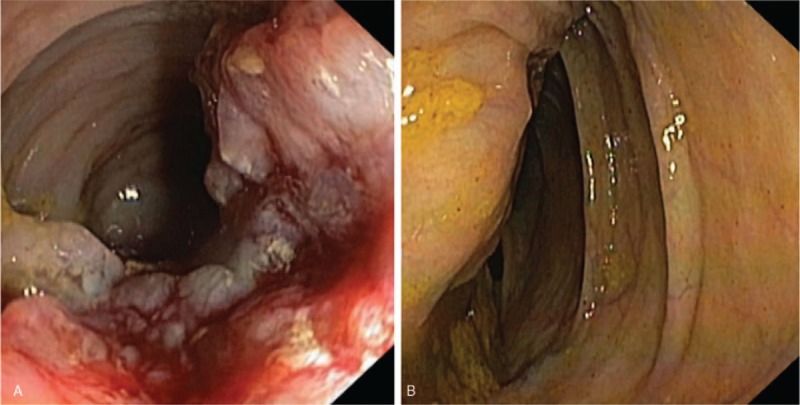
Colonoscopy. (A) Endoscopic view of the rectum's tumor. (B) Extrinsic compression of the cecum by the mass.

Colonoscopy detected an unknown lesion, which occupied two-thirds of rectum's lumen. It was located about 10 cm from the anal verge and had an 8 cm cranial extension. The cecum's mucosa appeared regular with the presence of ab-extrinsic compression, which compromised the correct functioning of the ileocecal valve (Fig. 4)

At our Institute, the case was discussed by the multidisciplinary group, which indicated an eco-guided biopsy. The histologic report described mucoid material with necrosis and positive for pan-keratin and CDX2 stains, which was highly suspicious for colon adenocarcinoma.

**Figure 5:**
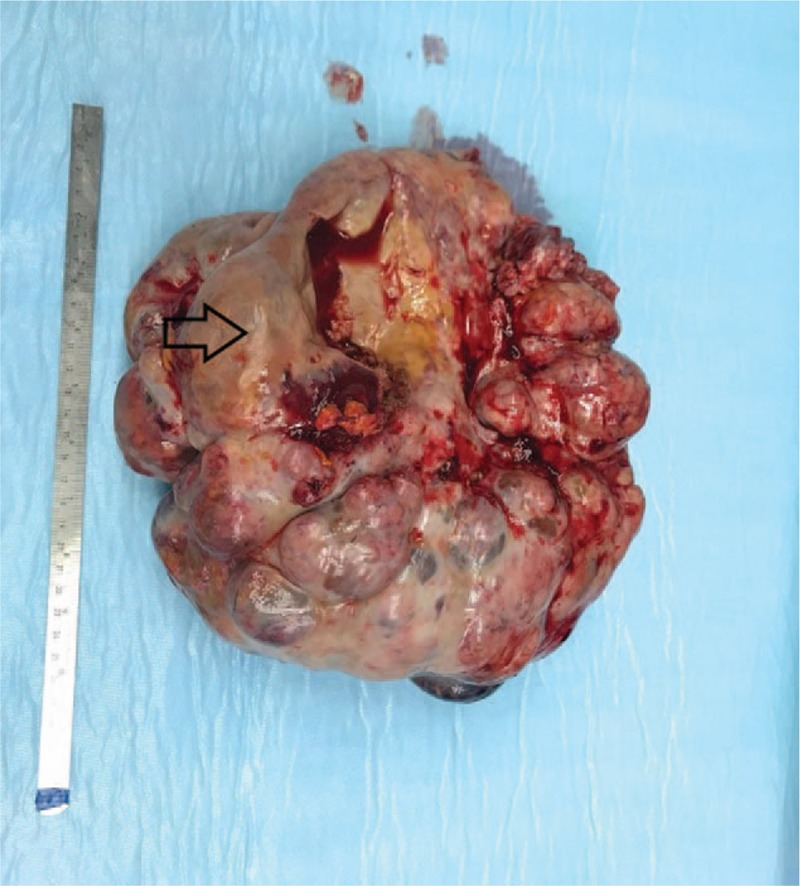
A 26-cm polycystic ovarian mass with areas containing mucin (white arrows).

The patient underwent surgery, where a midline incision was performed and a polycystic 26-cm lesion was found (Fig. 5). The lesion arose from the ovary and frozen section analysis revealed mucinous ovarian carcinoma. The omentum appeared to be involved by the disease, so it was removed. Against this background, we decided to perform a total hysterectomy with bilateral salpingo-oophorectomy and low anterior rectal resection with terminal colostomy. After surgery, the patient experienced good recovery and was discharged on the 8th postoperative day.

At the histopathological examination, the ovarian tumor was composed of a large amount of extracellular mucin containing malignant epithelial cells, which were arranged in well-differentiated glandular structures infiltrating the stroma. Although nondiagnostic, histopathology may be compatible with primitive mucinous ovarian carcinoma or adenocarcinoma from the gastrointestinal (GI) tract.

**Figure 6:**
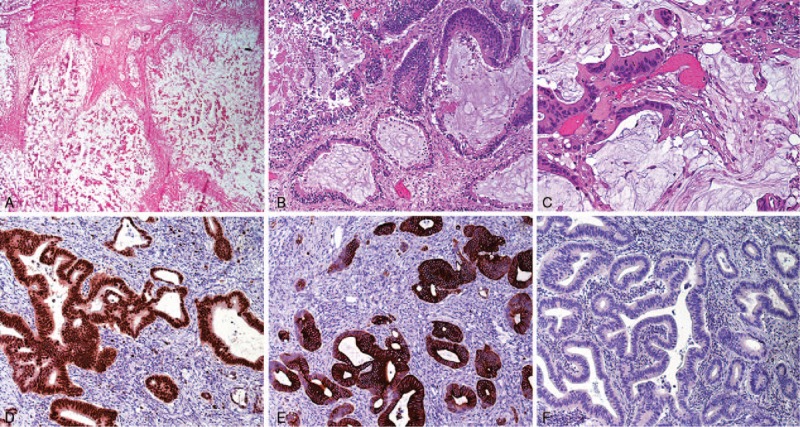
(A) Ovary section with the presence of a large amount of extracellular mucin containing malignant epithelial cells (frozen section H&E stain 4×). (B) Ovary section with well-differentiated glandular structures infiltrating the stroma (frozen section H&E stain 4×). (C) Rectum section with malignant glandular structures immerses in extracellular mucin (H&E stain 4×). (D) CDX2-positive immunohistochemical stain 20×. (E) CK20-positive immunohistochemical stain 20×. (F) Pax8-negative immunohistochemical stain 20×.

In rectal neoplasm, we found malignant glandular structures in a large amount of extracellular mucin. We performed immunohistochemical staining and found positive CDX2 stain. It represents a nuclear transcription factor that is critical for intestinal embryonic development and is relatively specific for intestinal epithelium. We also found positive CK20—an epithelial marker expressed in lower GI epithelium track; we did not find any stain for Pax8—a member of the paired box PAX family of transcription factors genes expressed in the Müllerian tract, and thyroid, parathyroid, renal, and thymic tumors (Fig. 6).

Interpretation of immunohistochemical panel in addition to routine hematoxylin and eosin (H&E) slide and clinicopathologic correlation allowed us to identify rectum as the primary site of the tumor, having a T4bN1b pathologic stage with vascular and lymphatic invasion.

The patient has a c.2194GA genotype, which results in a reduction of the DPD enzymatic activity for fluoropyrimidine; she has mutated BRAF and KRAS wild-type. Therefore, we started a 6-month first-line FOLFOX plus panitumumab adjuvant chemotherapy with a 50% reduced dosage, due to DPD mutation.

During this 10-month follow-up period and 8 months after surgery, radiologic tests confirmed the pelvic progression of the disease. The multidisciplinary group indicated a second-line chemotherapy protocol, which is still in progress.

## 3 Discussion

This case demonstrated particularly complex, as imaging and histology did not support us in reaching a clear preoperative diagnosis. Different studies, which tried to assess the clinical features of ovarian metastases from colorectal cancer, considered abdominal pain as the most frequent symptom, followed by bowel habit change and abdominal distension while bleeding per rectum turned out less frequent.^[3,8]^

Ovarian unilateral involvement was reported in 40% cases, even though the metastatic ovarian disease was more frequently bilateral and multinodular, in contrast to primary ovarian tumors that are usually unilateral. As regarding ovarian metastases, colon-located tumors metastasize more frequently than rectal ones do, as they tend to metastasize to the liver and lungs. Synchronous ovarian metastases are more frequent than metachronous ones. A higher (III or IV) T-stage disease leads ovarian metastasis to develop more frequently in young patients in comparison to older ones.^[3,4,8]^ According to such findings, our patient was very likely affected by a primary ovarian tumor.

Histologic features of pseudo-endometroid metastases from large intestine include: garland and cribriform histologic growth patterns; abundant intraluminal “dirty” necrosis; segmental destruction of glands; and absence of squamous metaplasia. Papillary architecture or mucinous carcinoma features can be found in nearly one-third of cases.^[3,4]^

Immunohistochemistry, on the contrary, can be very helpful in the diagnosis of ovarian involvement by metastatic colorectal cancer. Villin and CK20 are expressed in colorectal cancer metastatic to the ovary. CDX2 protein was expressed uniformly in almost all primary colonic adenocarcinomas, including 82% mucinous variants which showed strong nuclear staining. Ninety per cent of metastatic colonic adenocarcinomas expressed MUC2, but none expressed MUC5AC. On the contrary, 100% primary mucinous cystadenomas, borderline tumors, and carcinomas were positive for MUC5AC. Ca125 cancer antigen is present in only 4% to 15% of colorectal carcinomas. In another study, CEA was found to be elevated in 54.7% of patients.^[3,4,8]^ Our patient turned out positive for both CDX2 and pan-keratin. These findings gave us the first indication of possible colorectal origin. For this reason, we are deeply convinced that further studies on immunohistochemistry of ovarian metastases from colorectal cancer could be very helpful in diagnosing difficult cases. Erroi et al^[9]^ proposed a semiquantitative immunohistochemical staining score, where CK7−/CK20+ tumors were classified as ovarian metastases from colorectal cancer and CK7+/CK20− were considered as primary ovarian carcinoma.

Regarding computed tomography (CT) scan, bilateral complex ovarian masses showing smooth tumor margins and predominantly cystic features are more suggestive for ovarian metastases than primary ovarian tumors.^[10]^

A simple algorithm based on tumor size and laterality was suggested to tell primary ovarian tumors from metastatic ones. According to it, bilateral tumors of any size or <10 cm unilateral tumors are metastatic, whereas ≥10 cm unilateral tumors are primary ones. According to Yemelyanova et al, metastatic colorectal carcinomas behave as algorithm violators; as such tumors show up as large unilateral metastases which simulate primary ovarian tumors.^[3,5]^ Our patient falls into this category, with a unilateral gross mass that resulted positive for ovarian carcinoma at the frozen section. We know that in such cases frozen sections might be hardly interpreted, not always being able to lead us to a correct diagnosis.^[3]^

As far as treatment is concerned, we had no certain indication. We decided to perform cytoreductive surgery with excision of the primary tumor by low anterior rectal resection, terminal colostomy, and complete hysterectomy with bilateral salpingo-oophorectomy, as literature advised. Indication for bilateral oophorectomy even in unilateral involvement was dictated by the late onset of metachronous metastasis to the contralateral ovary.^[11]^ In the presence of evident metastatic disease which affects just the ovaries and has no peritoneal involvement, median survival is 61 months compared with 17-month median survival in women affected by concomitant peritoneal disease.^[6,12]^ The Japanese guidelines for colorectal cancer treatment recommend surgery whenever primary and metastatic lesions are fully resectable.^[13,14]^ Prophylactic oophorectomy during rectal surgery has a much more controversial role, considering that such procedure bears oncological advantages in premenopausal women, as they might not be prone to face the consequences of such procedure. On the contrary, menopausal women could often ask for the procedure, even though the benefits in this group are minor.^[15,16]^ A combination of cytoreductive surgery and chemotherapy might increase the overall survival, especially in extra-ovarian metastases. It is widely acknowledged that ovarian metastases are less responsive to chemotherapy, as ovaries may act as a “sanctuary site” that presents a favorable microenvironment for tumor growth.^[17]^

The presence of ovarian metastatic disease is a marker of systemic involvement and represents a negative prognostic factor, in addition to lymphovascular invasion, combined metastases, and bilateral ovarian metastases. In difficult cases, even the evaluation of permanent sections can give a misdiagnosis, leading to incorrect oncological treatment.^[8,15]^

## 4 Conclusions

Differential diagnosis between primary ovary tumors and colorectal ovarian metastases can be challenging, in particular, as preoperative biopsies are not often diagnostic. This situation leads the patient to the operating room without a precise diagnosis making it difficult to decide in advance which type of surgery to perform.

In ovarian masses that lack precise diagnosis, we are firmly convinced that colonoscopy is necessary to rule out the plausible colorectal origin.

Nonetheless, in many cases, surgery is not only therapeutic, but the only way to reach a precise assessment of the nature of ovarian mass. Only following final histopathologic diagnosis, appropriate systemic therapy can be carried out.

Immunohistochemical staining is paramount in diagnosing ovarian metastatic tumors. We deeply encourage further studies on this issue, to set up a well-defined immunohistochemical diagnostic panel.

## Acknowledgment

The authors thank Daniela Masi, MD, for English editing.

## Author contributions

**Conceptualization:** Carolina Castro Ruiz, Erica Pavesi, Maurizio Zizzo, Lara Ugoletti, Valerio Annessi.

**Data curation:** Federico Biolchini, Carolina Castro Ruiz, Giovanni Musci, Erica Pavesi, Maurizio Zizzo.

**Formal analysis:** Carolina Castro Ruiz, Giovanni Musci, Maurizio Zizzo.

**Funding acquisition:** Carolina Castro Ruiz, Erica Pavesi.

**Investigation:** Lara Ugoletti.

**Methodology:** Federico Biolchini, Carolina Castro Ruiz, Valerio Annessi.

**Software:** Federico Biolchini, Giovanni Musci, Maurizio Zizzo.

**Supervision:** Federico Biolchini, Giovanni Musci, Erica Pavesi, Valerio Annessi.

**Validation:** Federico Biolchini, Carolina Castro Ruiz, Giovanni Musci, Maurizio Zizzo, Lara Ugoletti, Valerio Annessi.

**Visualization:** Federico Biolchini, Carolina Castro Ruiz, Maurizio Zizzo, Lara Ugoletti, Valerio Annessi.

**Writing – original draft:** Federico Biolchini, Carolina Castro Ruiz, Giovanni Musci, Erica Pavesi, Maurizio Zizzo, Lara Ugoletti.

**Writing – review & editing:** Federico Biolchini, Carolina Castro Ruiz, Giovanni Musci, Erica Pavesi, Maurizio Zizzo, Lara Ugoletti, Valerio Annessi.

Carolina Castro Ruiz orcid: 0000-0002-1594-1853.
